# Cost-effectiveness of asenapine in the treatment of bipolar disorder in Canada

**DOI:** 10.1186/1471-244X-14-16

**Published:** 2014-01-22

**Authors:** Jean Lachaine, Catherine Beauchemin, Karine Mathurin, Dominique Gilbert, Maud Beillat

**Affiliations:** 1 Faculty of Pharmacy, University of Montreal, Station Centre-ville, PO Box 6128, H3C 3 J7 Montreal, Quebec, Canada; 2 Market Access and Health Outcomes, Lundbeck Canada Inc., Montreal, Quebec, Canada; 3 Health Economics and HTA, Lundbeck S.A.S., Issy-Les-Moulineaux, France

**Keywords:** Asenapine, Bipolar disorder, Antipsychotic, Canada, Cost-utility, Cost-effectiveness, Olanzapine

## Abstract

**Background:**

Bipolar disorder (BPD) is prevalent and is associated with a significant economic burden. Asenapine, the first tetracyclic antipsychotic approved in Canada for the treatment of BPD, has shown a comparable efficacy profile to other atypical antipsychotics. In addition, it is associated with a favourable metabolic profile and minimal weight gain potential. This study aimed to assess the economic impact of asenapine compared to olanzapine in the treatment of BPD in Canada.

**Methods:**

A decision tree combined with a Markov model was constructed to assess the cost-utility of asenapine compared with olanzapine. The decision tree takes into account the occurrence of extrapyramidal symptoms (EPS), the probability of switching to a different antipsychotic, and the probability of gaining weight. The Markov model takes into account long-term metabolic complications including diabetes, hypertension, coronary heart diseases (CHDs), and stroke. Analyses were conducted from both a Canadian Ministry of Health (MoH) and a societal perspective over a five-year time horizon with yearly cycles.

**Results:**

In the treatment of BPD, asenapine is a dominant strategy over olanzapine from both a MoH and a societal perspective. In fact, asenapine is associated with lower costs and more quality-adjusted life years (QALYs). Results of the probabilistic sensitivity analysis indicated that asenapine remains a dominant strategy in 99.2% of the simulations, in both a MoH and a societal perspective, and this result is robust to the many deterministic sensitivity analyses performed.

**Conclusions:**

This economic evaluation demonstrates that asenapine is a cost-effective strategy compared to olanzapine in the treatment of BPD in Canada.

## Background

Bipolar disorder (BPD), also known as manic-depressive disorders, is a chronic mood disorder. BPD type I is the classical syndrome, whereas BPD type II is characterized by more major depressive episodes [1]–[3]. Mental Health and Well-Being survey estimated the lifetime prevalence rate for BPD in Canada at 2.2%, which means that over 500,000 Canadians suffer from this condition [4]. In addition, the prevalence of suicide is high in the BPD population, with a lifetime prevalence of suicide attempts at up to 30% in 1996 in an American context [5].

Because BPD is one of the leading causes of disability, especially in active populations, it imposes a substantial economic burden on society [6]. An analysis of US epidemiological data collected between 1991 and 2009 estimated the direct and indirect costs of BPD I and II disorder at $US30.7 and $US120.3 billion, respectively, for a total economic burden of $US151.0 billion. From a European perspective, the estimated UK national cost of BPD disorder was £4.59 billion in 2007 pounds (about $US7.5 billion), with hospitalization during acute episodes accounting for the largest component of direct costs [7]. Moreover, BPD may also cause distress for caregivers and family members [8].

Mood stabilizers such as lithium, valproate, and carbamazepine are crucial in the treatment of BPD, however, the efficacy and tolerability of these agents is often inadequate, requiring the addition of other medications [3]. Atypical antipsychotics have shown efficacy in mania and depression, both as monotherapy or in combination with mood stabilizers, but are associated with substantial weight gain and metabolic changes, including extrapyramidal side effects (EPS) [9,10]. In addition to the significant impact of weight gain on quality of life and utility, the metabolic effects associated with atypical antipsychotics increase the risk of developing long-term complications. Furthermore, metabolic effects may constitute one of the many factors that contribute to medication nonadherence in patients with BPD.

To date, few economic evaluations have focused on the use of atypical antipsychotics in the treatment of BPD. Most economic evaluations concerning BPD were cost-effectiveness analysis, with the number of acute mood episodes avoided as the primary clinical outcome. Those economic evaluations have largely been based on Markov models, where treatment discontinuation or switches were taken into account [11]–[15]. To date, the use of asenapine for treating BPD has not been evaluated in Canada from an economic standpoint. Therefore, the objective of this study was to assess the economic impact of asenapine compared with olanzapine in the treatment of BPD over a 5-year time horizon.

## Method

For this economic evaluation, a model based cost-utility analysis was performed. For the base-case analysis, asenapine was compared to olanzapine because it has been used as the comparator in pivotal clinical trials [16]–[19]. In addition, olanzapine is one of the most commonly prescribed atypical antipsychotic drugs in Canada for BPD. The patient population presented the characteristics of patients in pivotal clinical trials of asenapine in BPD (moderate to severe BPD, onset at age of 40 years) [16]–[19]. To encompass short-term and long-term outcomes and the costs associated with atypical antipsychotic use, this economic evaluation was conducted over a five-year time horizon. In addition, a ten-year time-horizon was considered in a complementary analysis. Given the low adherence to antipsychotic medications in BPD patients, a longer perspective was not considered [20].

### Model structure

**Figure 1:**
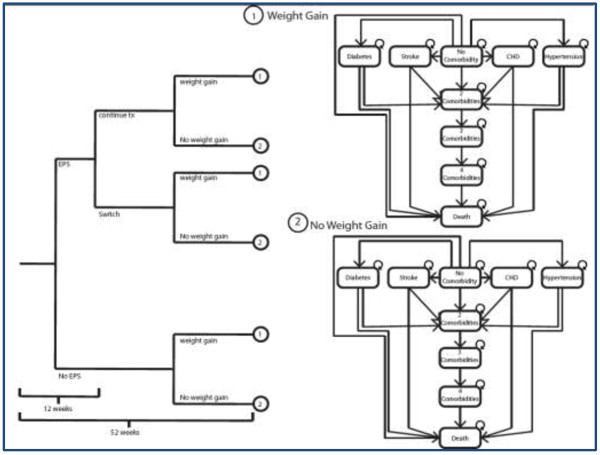
Model structure.

A decision tree combined with a time-dependent Markov model was constructed to assess the cost-effectiveness of asenapine compared with olanzapine in the treatment of BPD (Figure 1). A focus was placed on weight gain and long-term metabolic complications associated with treatments. According to expert clinicians (psychiatrists from the Douglas Mental Health University Institute, Montreal, Quebec), this model structure was clinically meaningful for accurate representation of disease evolution and treatment.

First, a decision tree with a one-year time horizon was constructed to take into account the occurrence of EPS-related events, the probability of switching treatment, and the probability of gaining weight. A proportion of patients who experienced EPS discontinued their first treatment and switched to another. The treatment used when a treatment switch occurred was aripiprazole, as it is the most recently approved atypical antipsychotic for schizophrenia (SCZ) and BPD indications, with similar metabolic properties to asenapine. Moreover, according to clinical experts, aripiprazole was considered the most appropriate option in case of treatment switch. Weight gain was considered according to results of pivotal clinical trials, where patients tend to significantly gain weight (≥7%) within the first year of treatment [16]–[19].

A Markov model was developed for the subsequent years of treatment. Markov health states included long-term metabolic complications such as diabetes, hypertension, CHDs, and stroke associated with weight gain, and the absorbing health state was death. According to the prevalence of each complication at age 40, as reported for the overall population, a proportion of BPD patients who entered the Markov model were already suffering from metabolic complications. Thereafter, patients progressed in the model health states with the reported annual incidence rate for each complication, taking into account the elevated risks for patients with weight gain associated with their BPD treatment. Diabetes and hypertension are chronic diseases and stroke and CHDs are punctual events with chronic consequences. Then, they were included in the model once the condition occurred, and they remained until death. It was assumed that complications could occur at any time during the horizon of the Markov model. In addition, although patients could acquire more than one complication during the model time period, it was assumed that only one complication could occur per patient per year. Further details on transition probabilities of the Markov model are available in Additional file 1.

### Clinical data

For the base-case scenario, the incidence rates of EPS-related adverse effects and of significant weight gain (≥7%) with asenapine and olanzapine were taken from pivotal clinical trials that directly compared the two treatments (Table 1) [16]–[19]. The proportion of treatment switch due to an EPS-related event in patients who experienced an EPS was assumed to be the same as that estimated in SCZ from clinical trials comparing asenapine and olanzapine because no data was available in BPD clinical trials [35]. A ≥7% weight gain was considered significant, according to data reported in pivotal asenapine clinical trials [16]–[19]. Moreover, the 7% weight gain is one of the clinical meaningful cuts used in most BPD clinical trials, and is recognized by the National Institute for Clinical Excellence (NICE) [36]–[39]. In order to appropriately capture weight gain complications, incidence data was taken from a study reporting on weight gain incidence after 52 weeks of antipsychotic treatment [19]. Because the EPS-related events occur early after treatment initiation, all pivotal studies, including short duration study, which reported a proportion of patients who experienced an EPS-related event, were considered.

**Table 1:** Model inputs: clinical parameters

	**Base-case**		**Lower bound**		**Upper bound**	
**Target population**[16]–[19]						
Age at onset	40		20		N/A	
**Incidence of adverse effects**[18]						
Significant weight gain						
Asenapine	39.2%		29.4%		49.0%	
Olanzapine	55.1%		41.3%		68.9%	
EPS-related event						
Asenapine	10.7%		7.2%		14.9%	
Olanzapine	9.5%		6.8%		13.1%	
**Risks of developing complications (OR)**						
	Men	Women	Men	Women	Men	Women
Diabetes [21,22]	2.69	1.9	2.17	1.5	3.34	2.3
Hypertension [23,24]	1.68	1.56	1.45	1.48	1.94	1.64
CHDs [22,25]	1.68	1.25	1.13	1.01	2.5	1.55
Stroke [26]	1.02	1.02	1.01	1.01	1.03	1.03
**Mortality risks**	Men	Women	Men	Women	Men	Women
Diabetes [27]	1.88	1.88	1.55	1.55	2.27	2.27
Hypertension [28]	1.44	1.34	1.00	1.00	2.88	2.68
CHDs [29,30]	2.20	1.60	2.00	1.20	2.40	2.10
Stroke [31]	2.37	2.37	2.11	2.07	2.64	2.70
**Utilities/Disutilities**						
*Disease-related*[32]						
BPD	0.800		0.580		1.000	
Weight gain in BPD	-0.066		-0.050		-0.083	
EPS in BPD	-0.074		-0.090		-0.053	
Weight of additional disutilities	0		0.5		1.0	
*Complications*[33]	Men	Women	Men	Women	Men	Women
Type II diabetes	-0.06	-0.05	-0.08	-0.08	-0.03	-0.03
Hypertension	-0.02	0	-0.03	-0.02	0	0.01
CHDs (Heart disease)	-0.07	-0.06	-0.09	-0.08	-0.05	-0.03
Effects of stroke	-0.17	-0.18	-0.23	-0.25	-0.12	-0.10
**Suicide rate**[34]	15.50		12.25		18.44	

Because data for the BPD population specifically was not available, the risks for selected complications in the general population who gained weight were extracted. The risks of developing long-term complications according to metabolic changes were extracted from the literature (Table 1). In studies that presented weight gain measures other than a ≥ 7% increase (weight gained (kg), body mass index change, etc.), the risk corresponding to the most conservative significant weight gain was applied. In fact, according to the average weight observed in BPD clinical trials, [16]–[19] an increase of at least 5 kg corresponds to a 7% increase of initial body weight.

### Costs data

All costs are expressed in Canadian dollar 2011 value. All costs estimated before 2011 were adjusted to June 2011 levels based on the health component of the Canadian Consumer Price Index [40].

The costs included in the analysis from a Ministry of health (MoH) perspective were those associated with medication, EPS management and healthcare resources used in the management of metabolic complications (Table 2). The cost of Saphris® was provided by Lundbeck Canada Inc., and the costs of the other antipsychotics were taken from the *Liste des médicaments* (list of medications) provided by the *Régie de l’assurance maladie du Québec* (RAMQ, Quebec’s health insurance board) [41]. For olanzapine, costs differ across dosages as well as manufacturers. Therefore, the RAMQ database was used to estimate the mean cost of olanzapine, based on its use in a real-life setting. First, patients with a diagnosis of BPD (International Classification of Diseases, ICD9 296.0-296.9) and who had a valid prescription for any dose and any brand of olanzapine on February 1, 2011 were identified. A mean daily cost was then estimated by multiplying the number of tablets used per day by the unit cost of olanzapine. For aripiprazole used in cases of treatment switch, the unit cost in the *Liste des médicaments* was directly multiplied by the number of tablets recommended daily, because it is commercialized under the original brand only and all available doses are the same price. According to expert clinicians as well as the Canadian guidelines, management of EPS-related symptoms would require one extra physician visit, as these symptoms can usually be attenuated by reducing the dosage or switching the medication [48]. The costs associated with metabolic complications were those covered by the MoH, including medical costs (physician, outpatient care, emergency visits, hospitalizations, and intensive care unit) and medications. Costs associated with a complication were estimated using data from pharmaceutical and medical services retrieved from Quebec’s Provincial Health Plan database. The RAMQ data are anonymized and are publicly available, at a cost, for research purposes. The RAMQ Direction for analysis and management of information manage the distribution of RAMQ data to external parties. More specifically, for each complication, the difference between the median annual costs incurred by patients aged from 40 to 44 years who had the complication from January 1, 2003 to December 31, 2009 and the median annual costs incurred by patients in the same age range who did not have the complication was calculated.

**Table 2:** Model inputs: costs

	**Event cost ($)**		
	**Base-case**	**Lower bound**	**Upper bound**
**Treatment Costs**			
Cost of annual treatment			
Asenapine	1,030.00	N/A	N/A
Olanzapine [41]	1,871.00	1,316.00	2,426.00
-Cost of EPS management [42]	60.00	0.00	N/A
**Costs associated with long-term metabolic complications**			
Direct costs*			
Diabetes	3,834.77	1,215.00	17,072.00
Hypertension	571.00	233.00	827.00
CHDs			
Fatal CHDs	7,093.20	775.00	52,617.00
CHDs (Year 1)	2,481.24	818.00	8,819.00
CHDs (Year 2–5)	1,146.11	360.00	5,795.00
Stroke			
Fatal stroke	30,776.93	7,362.00	34,165.00
Stroke (Year 1)	4,034.86	1,395.00	10,560.00
Stroke (Year 2–5)	1,867.59	452.00	8,692.00
*Productivity losses*[28,43]–[46]			
Diabetes	528.00	396.00	660.00
Hypertension	119.00	89.25	148.75
CHDs	3,109.00	2,331.75	3,886.25
Stroke	4,322.00	3,241.50	5,402.50
*Informal care*[47]			
Stroke	3,770.00	2,827.50	4,712.50

*Estimated from the RAMQ database.

For the analysis using a societal perspective, additional costs associated with loss of productivity and informal care due to long-term metabolic complications were also considered (Table 2). Costs associated with productivity losses were obtained from Canadian public sources [28,43]–[46]. The estimated overall productivity loss for a complication was divided by the prevalence of the complication in the overall Canadian population in the estimated year to obtain the cost per patient. When prevalence data for the year of estimation were unavailable, the prevalence in the nearest available year was used. In the literature, only patients with stroke have been reported to require significant home care [49]. In fact, based on a Canadian study by Goeree et al., caregiver expenses account for 12% of the total one-year stroke cost, which amounts to $27,245 for a person younger than 55 years [47]. Therefore, in the present economic evaluation, only informal care associated with stroke was considered.

### Utility

Utilities associated with BPD and disutilities associated with EPS and metabolic complications were taken into account in this analysis (Table 1). Revicki and colleagues used the standard gamble method to estimate the utilities for BPD [32]. They found that BPD was associated with a mean utility of 0.80. They also found that weight gain was associated with a 0.066 decrease in utility. Because the disutilities associated with EPS were unavailable for BPD, it was assumed that the disutility associated with EPS in BPD was similar to that for SCZ. Therefore, a 0.074 mean reduction in utility for acute EPS events, as reported in Lenert et al.’s SCZ study, was included in the model [50]. Disutilities associated with weight gain and EPS were subtracted from the baseline utility observed in BPD patients [39]. EPS-associated disutility was estimated to last for three months, whereas it was permanent in the case of weight gain.

Long-term metabolic complications of interest are also associated with a significant reduction in utility. Schultz and Kopec estimated the impact of various self-reported chronic conditions on health-related quality of life, as measured by the Health Utilities Index 3 [33]. According to their results, the mean disutility values for each metabolic complication included in the model were taken into account. (Table 1) When more than one complication was present concomitantly, the disutility of only the most debilitating complication was considered in the base-case analysis. Taking into account baseline utility for BPD, these disutility values were used to adjust the number of quality-adjusted life years (QALYs) according to development of long-term metabolic complications.

### Mortality

Survival rates were taken from the most recent Canadian life tables available for men and women for the general population [51]. Because BPD patients present higher suicide rates than the general population, mortality rates of the general population were adjusted by suicide rates reported in BPD patients. According to a meta-analysis by Harris et al., the suicide rate was 15.5 times higher in a BPD population compared to the general population (Table 1) [34]. To incorporate the higher risk of suicide associated with BPD in the model, the estimated mortality due to suicide in the general Canadian population for men and women at 40 years was first subtracted from the mortality observed in the general population [52]. The higher risk of suicide in the BPD population was then added.

The risks of mortality associated with complications of interest were also taken into account (Table 1). In the case of several concomitant complications, all mortality risks were included for the base-case scenario. The risk of mortality caused by fatal stroke or CHD events (mostly myocardial infarction) were also included. Fatal cases of stroke and CHD events were estimated using the RAMQ database.

### Analyses

The incremental cost-utility ratios (ICURs) were calculated as the total cost associated with asenapine treatment minus the total cost associated with olanzapine treatment divided by the number of QALYs associated with asenapine minus the number of QALYs associated with olanzapine. Consistent with the Canadian Agency for Drugs and Technologies in Health recommendations, costs and benefits were discounted at a rate of 5% per year.

A complementary analysis using a ten-year time horizon was performed to assess the impact of metabolic complications over a longer period.

Robustness of the results of this analysis was tested by deterministic and probabilistic sensitivity analyses. Confidence intervals were used as lower and upper bounds when available. When confidence intervals were not available, a ±25% variation was applied to the base-case parameters (Table 1). Deterministic analyses were performed by varying individually within lower and upper bounds the gender distribution, starting age, incidence of adverse effects, risks of developing selected long-term metabolic complications, mortality risks, costs, utilities, and suicide and discount rates. Probabilistic sensitivity analyses were conducted using Monte Carlo simulation by varying simultaneously baseline characteristics, clinical outcomes, resource utilization and cost parameter inputs. The probabilistic analysis was undertaken by randomly sampling each parameter distributions and calculating the expected costs and expected number of QALYs for that combination of parameter values for a total of 10,000 replications. Probabilistic sensitivity analysis was performed using Oracle Crystal Ball version 11.1.1.1.00 to assess the overall impact of uncertainty associated with the study parameters on the base-case results.

## Results

### Base-case analysis

Over a five-year time period, asenapine was found to be a dominant strategy over olanzapine in the treatment of BPD, from both a MoH and a societal perspective (Table 3). Thus, the costs associated with the use of asenapine are lower than those associated with the use of olanzapine, and the number of QALYs obtained with asenapine is higher than the number obtained with olanzapine. Per 1,000 patients, there was a gain of 84.84 QALY with the use of asenapine and a decrease in cost of Can$3,847,300 from a MoH perspective and a decrease of Can$3,878,343 from a societal perspective.

**Table 3:** ICURs – base-case scenario and ten-year time horizon scenario / 1,000 individuals

	**Costs ($)**	**Incremental costs ($)**	**QALYs**	**Incremental QALYs**	**ICUR ($/QALY)**
***MoH perspective***					
Base-case scenario					
Olanzapine	9,729,158	-3,847,300	3,301	84.84	dominant
Asenapine (Saphris®)	5,881,858		3,386		
Ten-year time horizon scenario					
Olanzapine	18,233,001	-6,956,705	5,784	160.67	dominant
Asenapine (Saphris®)	11,276,296		5,945		
***Societal perspective***					
Base-case scenario					
Olanzapine	11,466,846	-3,878,343	3,301	84.84	dominant
Asenapine (Saphris®)	7,588,702		3,386		
Ten-year time horizon scenario					
Olanzapine	22,527,000	-7,131,099	5,784	160.67	dominant
Asenapine (Saphris®)	15,395,901		5,945		

### Complementary analyses

From both MoH and societal perspectives, asenapine remained a dominant alternative over a ten-year time horizon (Table 3).

### Sensitivity analysis

Results of the deterministic and probabilistic analyses confirmed the robustness of the base-case results. According to the deterministic analysis results, asenapine remained a dominant strategy from both perspectives. The individual variation of each parameter had no impact on the base-case results. The probabilistic sensitivity analysis also confirmed the robustness of the base-case results. From both a MoH and a societal perspective, asenapine was a dominant alternative over olanzapine in 99.2% of the Monte Carlo simulations.

## Discussion

This economic evaluation suggests that, compared with olanzapine, asenapine is a dominant alternative from both a MoH and a societal perspective. Asenapine is associated with a lower treatment cost and a lower risk of gaining weight (39.2% vs. 55.1%, respectively) than olanzapine, which leads to a lower risk of developing metabolic complications. Moreover, the results of the exhaustive sensitivity analysis confirmed the robustness of the base-case results.

This is the first Canadian economic evaluation of asenapine in the treatment of BPD. To date, few studies have assessed the economic impact of atypical antipsychotics with a focus on metabolic changes and their complications on quality of life and survival in SCZ and BPD context. For example, in a recent study by McIntyre et al., a semi-Markov model was constructed to evaluate the cost and predicted incidence of long-term complications (type-2 diabetes, angina, myocardial infarction, stroke, and cardiovascular-related deaths) associated with metabolic changes induced by treatment with atypical antipsychotic agents in SCZ [53]. More recently, Kasteng et al. developed a Markov health state transition model and found that treatment with aripiprazole was a dominant strategy over olanzapine in the treatment of SCZ and BPD, with 0.08 QALYs gained and cost savings of $US4,000 per patient over a lifetime horizon [15]. However, this study has some limitations, including the lack of consideration of non-metabolic adverse events and drug switching or discontinuation.

This economic evaluation has several strengths. First, the type of analysis chosen, a cost-utility analysis, allows considering atypical antipsychotic-related metabolic effects on mortality and morbidity. In addition, the analysis accounted for adverse events associated with treatment, including EPS and weight gain as well as treatment switches due to EPS. Furthermore, because weight gain is a progressive adverse effect, the choice of a one-year period for weight gain development better reflects the reality. Pharmaceutical and medical services were taken from a RAMQ database to estimate the costs of metabolic complications based on real-life settings. The RAMQ database was also used to accurately estimate the cost of antipsychotics used by BPD patients in real-life settings. Because different doses of antipsychotics are used for different indications, estimates of treatment costs in real-life settings, and specifically in a BPD population, constituted the most appropriate method.

However, this economic evaluation has several limitations. First, despite the limited clinical data on atypical antipsychotics in the treatment of BPD, there was sufficient evidence to develop an economic evaluation comparing asenapine to olanzapine specifically for this indication. However, there was insufficient evidence to support an economic evaluation comparing asenapine to other atypical antipsychotics. In addition, because certain data on BPD patients were unavailable, some model parameters, such as disutility associated with EPS and risks of developing complications, were obtained in SCZ patients and general population respectively. Moreover, given the absence of direct comparison between asenapine and olanzapine in clinical trials of antipsychotics used as combination therapy, this economic evaluation focused solely on a comparison of these agents as monotherapy for BPD.

Then, as for any model-based analysis, many assumptions were made, which may increase the uncertainty of the results. However, a conservative approach was adopted to define each model assumption. For example, in the base-case analysis, the disutility of only one complication was taken into account, even when patients had more than one complication. In addition, the time horizon was limited to five years, although the benefits of reducing weight gain can extend beyond that period. However, the impact of a 10-year time horizon was assessed in the complementary analyses. Moreover, the development of complications was limited to only one per cycle, although some patients may develop more than one complication in the same year. Furthermore, the model allows for only one treatment switch, although several switches may be required before obtaining the optimal treatment in terms of efficacy and safety. A further limitation of this analysis is the assumption that patients remained on their medication continuously for five years, even though studies have reported significant nonadherence rates to antipsychotic treatment across BPD populations [54,55]. However, lack of treatment persistence was observed across all atypical antipsychotic agents. Therefore, the predicted clinical and economic benefits with asenapine would apply for the proportion of BPD patients who would persist with their pharmacological regimen. In addition, this persistence assumption has been applied both to asenapine and olanzapine. Moreover, the model assumed that metabolic complications are independent consequences of weight gain. More specifically, the model included increased risks of metabolic complications due to weight gain, but did not take into account the impact of existing comorbidities on the development of new complications. For example, literature reported that people with type II diabetes have a risk of CHD 2–4 times greater than the general population [56,57]. However, people suffering from diabetes often presents other factors that could also be associated with the incidence of CHD. It is highly probable that there is a synergy between these factors and complications. It becomes difficult to isolate the risk of developing CHD associated with the combination of diabetes and weight gain. On the other hand, the addition of these risks would probably lead to an overestimation of the risk of developing CHD. Therefore, the risks of developing CHD and diabetes associated with weight gain were considered separately, as the model focuses on the metabolic effects associated with antipsychotic use. The choice of including the risk of developing CHD when suffering from diabetes would possibly lead to a more favourable cost-effectiveness ratio for asenapine. However, when increasing the risk of developing CHD in sensitivity analyses, asenapine remains a dominant strategy over olanzapine. The assumption of considering metabolic complications as independent outcomes was therefore conservative. Furthermore, the model did not allow for potential dosage reduction associated with EPS occurrence. In any case, this would have a minimal impact on the overall result, considering the relatively low incidence of EPS that requires dose adjustment in current practice. Despite these limitations, findings of this analysis are robust according to sensitivity analyses.

## Conclusion

In conclusion, this economic evaluation showed that asenapine is a dominant alternative over olanzapine in the treatment of BPD.

## Abbreviations

BPD: Bipolar disorder; CHD: Coronary heart disease; EPS: Extrapyramidal symptoms; ICUR: Incremental cost-utility ratio; MoH: Ministry of Health; QALY: Quality-adjusted life year; RAMQ: *Régie de l’assurance maladie du Québec*; SCZ: Schizophrenia.

## Competing interests

Dr Lachaine, Ms Beauchemin and Ms Mathurin received consulting fees from Lundbeck Canada Inc. At the time of study, Dominique Gilbert was an employee of Lundbeck Canada and Maud Beillat was an employee of Lundbeck S.A.S. The authors declare that they have no competing interests.

## Authors’ contributions

JL, CB, KM, MB, and DG participated in the design of the study. JL, CB and KM performed the analyses and drafted the manuscript. MB, and DG revised the manuscript. All authors read and approved the final manuscript.

## Authors’ information

JL, PhD, is associate professor in pharmacoeconomics at the Faculty of Pharmacy, University of Montreal. CB, MSc, is PhD student in pharmacoeconomics at the Faculty of Pharmacy, University of Montreal. KM, MSc, is research assistant at the Faculty of Pharmacy, University of Montreal. DG is Senior Director, Market Access and Health Outcomes at Lundbeck Canada. MB is Global Senior Health Economics and HTA Manager at Lundbeck S.A.S.

## Pre-publication history

The pre-publication history for this paper can be accessed here:

http://www.biomedcentral.com/1471-244X/14/16/prepub

## Supplementary Material

Additional file 1 **Transition rates for the Markov model.** This table provides the probability of developing each complication (hypertension, diabetes, CHD, stroke, fatal MI, fatal stroke) during the 5-year horizon time of the Markov model. Click here for file

## References

[B1] Bauer M Pfennig A Epidemiology of bipolar disorders Epilepsia 2005 46 Suppl 4 8 13 15968806 10.1111/j.1528-1167.2005.463003.x

[B2] Belmaker RH Bipolar disorder N Engl J Med 2004 351 5 476 486 10.1056/NEJMra035354 15282355

[B3] Culver JL Arnow BA Ketter TA Bipolar disorder: improving diagnosis and optimizing integrated care J Clin Psychol 2007 63 1 73 92 10.1002/jclp.20333 17115430

[B4] Schaffer A Cairney J Cheung A Veldhuizen S Levitt A Community survey of bipolar disorder in Canada: lifetime prevalence and illness characteristics Can J Psychiatry 2006 51 1 9 16 16491979 10.1177/070674370605100104

[B5] Oquendo MA Waternaux C Brodsky B Parsons B Haas GL Malone KM Mann JJ Suicidal behavior in bipolar mood disorder: clinical characteristics of attempters and nonattempters J Affect Disord 2000 59 2 107 117 10.1016/S0165-0327(99)00129-9 10837879

[B6] Murray CJ Lopez AD Global mortality, disability, and the contribution of risk factors: Global Burden of Disease Study Lancet 1997 349 9063 1436 1442 10.1016/S0140-6736(96)07495-8 9164317

[B7] Das Gupta R Guest JF Annual cost of bipolar disorder to UK society Br J Psychiatry 2002 180 227 233 10.1192/bjp.180.3.227 11872515

[B8] Laxman KE Lovibond KS Hassan MK Impact of bipolar disorder in employed populations Am J Manag Care 2008 14 11 757 764 18999910

[B9] Bowden CL Grunze H Mullen J Brecher M Paulsson B Jones M Vagero M Svensson K A randomized, double-blind, placebo-controlled efficacy and safety study of quetiapine or lithium as monotherapy for mania in bipolar disorder J Clin Psychiatry 2005 66 1 111 121 10.4088/JCP.v66n0116 15669897

[B10] Calabrese JR Keck PE Jr Macfadden W Minkwitz M Ketter TA Weisler RH Cutler AJ McCoy R Wilson E Mullen J A randomized, double-blind, placebo-controlled trial of quetiapine in the treatment of bipolar I or II depression Am J Psychiatry 2005 162 7 1351 1360 10.1176/appi.ajp.162.7.1351 15994719

[B11] Woodward TC Tafesse E Quon P Lazarus A Cost effectiveness of adjunctive quetiapine fumarate extended-release tablets with mood stabilizers in the maintenance treatment of bipolar I disorder Pharmacoeconomics 2010 28 9 751 764 10.2165/11538350-000000000-00000 20623994

[B12] Calvert NW Burch SP Fu AZ Reeves P Thompson TR The cost-effectiveness of lamotrigine in the maintenance treatment of adults with bipolar I disorder J Manag Care Pharm 2006 12 4 322 330 16792438 10.18553/jmcp.2006.12.4.322 PMC10437984

[B13] McKendrick J Cerri KH Lloyd A D'Ausilio A Dando S Chinn C Cost effectiveness of olanzapine in prevention of affective episodes in bipolar disorder in the United Kingdom J Psychopharmacol 2007 21 6 588 596 17050661 10.1177/0269881106068395

[B14] Fajutrao L Paulsson B Liu S Locklear J Cost-effectiveness of quetiapine plus mood stabilizers compared with mood stabilizers alone in the maintenance therapy of bipolar I disorder: results of a Markov model analysis Clin Ther 2009 31 Pt 1 1456 1468 19698903 10.1016/j.clinthera.2009.06.009

[B15] Kasteng F Eriksson J Sennfalt K Lindgren P Metabolic effects and cost-effectiveness of aripiprazole versus olanzapine in schizophrenia and bipolar disorder Acta Psychiatr Scand 2011 124 3 214 225 10.1111/j.1600-0447.2011.01716.x 21609324

[B16] McIntyre RS Cohen M Zhao J Alphs L Macek TA Panagides J A 3-week, randomized, placebo-controlled trial of asenapine in the treatment of acute mania in bipolar mania and mixed states Bipolar Disord 2009 11 7 673 686 10.1111/j.1399-5618.2009.00748.x 19839993

[B17] McIntyre RS Cohen M Zhao J Alphs L Macek TA Panagides J Asenapine versus olanzapine in acute mania: a double-blind extension study Bipolar Disord 2009 11 8 815 826 10.1111/j.1399-5618.2009.00749.x 19832806

[B18] McIntyre RS Cohen M Zhao J Alphs L Macek TA Panagides J Asenapine in the treatment of acute mania in bipolar I disorder: a randomized, double-blind, placebo-controlled trial J Affect Disord 2010 122 1–2 27 38 20096936 10.1016/j.jad.2009.12.028

[B19] McIntyre RS Cohen M Zhao J Alphs L Macek TA Panagides J Asenapine for long-term treatment of bipolar disorder: a double-blind 40-week extension study J Affect Disord 2010 126 3 358 365 10.1016/j.jad.2010.04.005 20537396

[B20] Johnson FR Ozdemir S Manjunath R Hauber AB Burch SP Thompson TR Factors that affect adherence to bipolar disorder treatments: a stated-preference approach Med Care 2007 45 6 545 552 10.1097/MLR.0b013e318040ad90 17515782

[B21] Oguma Y Sesso HD Paffenbarger RS Jr Lee IM Weight change and risk of developing type 2 diabetes Obes Res 2005 13 5 945 951 10.1038/oby.2005.109 15919849

[B22] Willett WC Manson JE Stampfer MJ Colditz GA Rosner B Speizer FE Hennekens CH Weight, weight change, and coronary heart disease in women Risk within the 'normal' weight range. JAMA 1995 273 6 461 465 10.1001/jama.1995.03520300035033 7654270

[B23] Williams PT Increases in weight and body size increase the odds for hypertension during 7 years of follow-up Obesity (Silver Spring) 2008 16 11 2541 2548 10.1038/oby.2008.396 18756262 PMC4108283

[B24] Huang Z Willett WC Manson JE Rosner B Stampfer MJ Speizer FE Colditz GA Body weight, weight change, and risk for hypertension in women Ann Intern Med 1998 128 2 81 88 10.7326/0003-4819-128-2-199801150-00001 9441586

[B25] Galanis DJ Harris T Sharp DS Petrovitch H Relative weight, weight change, and risk of coronary heart disease in the Honolulu Heart Program Am J Epidemiol 1998 147 4 379 386 10.1093/oxfordjournals.aje.a009460 9508105

[B26] Asplund K Karvanen J Giampaoli S Jousilahti P Niemela M Broda G Cesana G Dallongeville J Ducimetriere P Evans A Relative risks for stroke by age, sex, and population based on follow-up of 18 European populations in the MORGAM Project Stroke 2009 40 7 2319 2326 10.1161/STROKEAHA.109.547869 19520994

[B27] Egede LE Nietert PJ Zheng D Depression and all-cause and coronary heart disease mortality among adults with and without diabetes Diabetes Care 2005 28 6 1339 1345 10.2337/diacare.28.6.1339 15920049

[B28] Public Health Agency of Canada Report from the Canadian Chronic Disease Surveillance System: Hypertension in Canada, 2010 Chronic Disease Surveillance Division 2010 Ottawa: Public Health Agency of Canada 28 Available from: http://www.phac-aspc.gc.ca/cd-mc/cvd-mcv/ccdss-snsmc-2010/pdf/CCDSS_HTN_Report_FINAL_EN_20100513.pdf.

[B29] Lotufo PA Gaziano JM Chae CU Ajani UA Moreno-John G Buring JE Manson JE Diabetes and all-cause and coronary heart disease mortality among US male physicians Arch Intern Med 2001 161 2 242 247 10.1001/archinte.161.2.242 11176738

[B30] Biagini E Elhendy A Schinkel AF Rizzello V van Domburg RT Krenning BJ Schouten O Sozzi FB Branzi A Rocchi G Comparison of all-cause mortality in women with known or suspected coronary artery disease referred for dobutamine stress echocardiography with normal versus abnormal test results Am J Cardiol 2005 95 9 1072 1075 10.1016/j.amjcard.2004.12.061 15842974

[B31] Bronnum-Hansen H Davidsen M Thorvaldsen P Long-term survival and causes of death after stroke Stroke 2001 32 9 2131 2136 10.1161/hs0901.094253 11546907

[B32] Revicki DA Hanlon J Martin S Gyulai L Nassir Ghaemi S Lynch F Mannix S Kleinman L Patient-based utilities for bipolar disorder-related health states J Affect Disord 2005 87 2–3 203 210 16005983 10.1016/j.jad.2005.03.017

[B33] Schultz SE Kopec JA Impact of chronic conditions Health Rep 2003 14 4 41 53 14608795

[B34] Harris EC Barraclough B Suicide as an outcome for mental disorders A meta-analysis. Br J Psychiatry 1997 170 205 228 10.1192/bjp.170.3.205 9229027

[B35] Schoemaker J Naber D Vrijland P Panagides J Emsley R Long-term assessment of Asenapine vs Olanzapine in patients with schizophrenia or schizoaffective disorder. Pharmacopsychiatry 2010 43 4 138 146 20205074 10.1055/s-0030-1248313

[B36] Lieberman JA Stroup TS McEvoy JP Swartz MS Rosenheck RA Perkins DO Keefe RS Davis SM Davis CE Lebowitz BD Effectiveness of antipsychotic drugs in patients with chronic schizophrenia N Engl J Med 2005 353 12 1209 1223 10.1056/NEJMoa051688 16172203

[B37] McEvoy JP Lieberman JA Perkins DO Hamer RM Gu H Lazarus A Sweitzer D Olexy C Weiden P Strakowski SD Efficacy and tolerability of olanzapine, quetiapine, and risperidone in the treatment of early psychosis: a randomized, double-blind 52-week comparison Am J Psychiatry 2007 164 7 1050 1060 10.1176/appi.ajp.164.7.1050 17606657

[B38] Stroup TS Lieberman JA McEvoy JP Swartz MS Davis SM Rosenheck RA Perkins DO Keefe RS Davis CE Severe J Effectiveness of olanzapine, quetiapine, risperidone, and ziprasidone in patients with chronic schizophrenia following discontinuation of a previous atypical antipsychotic Am J Psychiatry 2006 163 4 611 622 10.1176/appi.ajp.163.4.611 16585435

[B39] National Collaborating Centre for Mental Health (UK) Schizophrenia: Core Interventions in the Treatment and Management of Schizophrenia in Primary and Secondary Care (Update) [Internet] 2009 Leicester (UK): British Psychological Society Mar. (NICE Clinical Guidelines, No. 82.) Available from: http://www.ncbi.nlm.nih.gov/books/NBK11681/20704054

[B40] Consumer Price Index, Health Care: CANSIM Table 326–0020

[B41] Régie de l'assurance maladie du Québec List of Medications 33 Last updated on 3 October 2013. Quebec?: Bibliothèque et Archives nationales du Québec, 2011. Available from: https://www.prod.ramq.gouv.qc.ca/DPI/PO/Commun/PDF/Liste_Med/Liste_Med/liste_med_2011_10_03_en.pdf

[B42] Régie de l'assurance maladie du Québec Manuel des médecins spécialistes 2011

[B43] Public Health Agency of Canada Tracking Heart Disease and Stroke in Canada 2009 Ottawa

[B44] Patra J Popova S Rhem J Bondy S Flint R Giesbrecht N Economic Cost of Chronic Disease in Canada: 1995–2003 2007

[B45] Heart and Stroke Foundation of Canada The Growing Burden of Heart Disease and Stroke in Canada 2003 2003 Ottawa, Canada In vol. 1-896242-30-8

[B46] Public Health Agency of Canada Diabetes in Canada:Highlights from the National Diabetes Surveillance System 2004 – 2005 2008 Ottawa: PHAC

[B47] Goeree R Blackhouse R Petrovic R Salama S Cost of stroke in Canada: a 1-year prospective study J Med Econ 2005 8 147 167 10.3111/200508147167

[B48] Canadian Psychiatric Association Clinical practice guidelines. Treatment of schizophrenia Can J Psychiatry 2005 50 13 Suppl 1 7S 57S 16529334

[B49] Wilkins K Park E Home care in Canada Health Rep 1998 10 1 29 37 ENG); 31-40(FRE 9836884

[B50] Lenert LA Sturley AP Rapaport MH Chavez S Mohr PE Rupnow M Public preferences for health states with schizophrenia and a mapping function to estimate utilities from positive and negative symptom scale scores Schizophr Res 2004 71 1 155 165 10.1016/j.schres.2003.10.010 15374583

[B51] Statistics Canada Life Tables, Canada, provinces and territories, 2000–2002 (Catalogue no. 84-537-XIE) 2006 Ottawa: Minister of Industry E

[B52] Statistics Canada Minister of Industry Death and mortality rates, by selected groupes causes, age, group and sex, Table 102–0551 (Catalogue no. 84F0209X) 2007 Ottawa

[B53] McIntyre RS Cragin L Sorensen S Naci H Baker T Roussy JP Comparison of the metabolic and economic consequences of long-term treatment of schizophrenia using ziprasidone, olanzapine, quetiapine and risperidone in Canada: a cost-effectiveness analysis J Eval Clin Pract 2010 16 4 744 755 10.1111/j.1365-2753.2009.01189.x 20545800

[B54] Correll CU Frederickson AM Kane JM Manu P Equally increased risk for metabolic syndrome in patients with bipolar disorder and schizophrenia treated with second-generation antipsychotics Bipolar Disord 2008 10 7 788 797 10.1111/j.1399-5618.2008.00625.x 19032710

[B55] Treuer T Hoffmann VP Chen AK Irimia V Ocampo M Wang G Singh P Holt S Factors associated with weight gain during olanzapine treatment in patients with schizophrenia or bipolar disorder: results from a six-month prospective, multinational, observational study World J Biol Psychiatry 2009 10 4 Pt 3 729 740 19606406 10.1080/15622970903079507

[B56] Koskinen P Manttari M Manninen V Huttunen JK Heinonen OP Frick MH Coronary heart disease incidence in NIDDM patients in the Helsinki Heart Study Diabetes Care 1992 15 7 820 825 10.2337/diacare.15.7.820 1516498

[B57] Manson JE Colditz GA Stampfer MJ Willett WC Krolewski AS Rosner B Arky RA Speizer FE Hennekens CH A prospective study of maturity-onset diabetes mellitus and risk of coronary heart disease and stroke in women Arch Intern Med 1991 151 6 1141 1147 10.1001/archinte.1991.00400060077013 2043016

