# Supplementary material for: Cost-effectiveness of asenapine in the treatment of bipolar disorder in Canada
Source: BMC Psychiatry. 2014 Jan 22;14:16. doi: 10.1186/1471-244X-14-16 (PMC3905654; doi:10.1186/1471-244X-14-16)
Supplement: Additional file 1 — Transition rates for the Markov model. This table provides the probability of developing each complication (hypertension, diabetes, CHD, stroke, fatal MI, fatal stroke) during the 5-year horizon time of the Markov model. [file 1471-244X-14-16-S1.docx]

| **Complications** | **Transition probabilities**  **Male** | **Transition probabilities**  **Female** | **Source of data** |
| --- | --- | --- | --- |
| Hypertension** ** | Year 0: 1.52%*  Year 1: 1.52%  Year 2: 1.52%  Year 3: 1.52%  Year 4: 1.52%  Year 5: 2.22% | Year 0: 1.32%*  Year 1: 1.32%  Year 2: 1.32%  Year 3: 1.32%  Year 4: 1.32%  Year 5: 2.03% | Public Health Agency of Canada |
| Diabetes** ** | Year 0: 0.58%*  Year 1: 0.58%  Year 2: 0.58%  Year 3: 0.58%  Year 4: 0.58%  Year 5: 0.85% | Year 0: 0.44%*  Year 1: 0.44%  Year 2: 0.44%  Year 3: 0.44%  Year 4: 0.44%  Year 5: 0.62% | Public Health Agency of Canada |
| CHD | Year 0: 0.18%*  Year 1: 0.18%  Year 2: 0.18%  Year 3: 0.18%  Year 4: 0.18%  Year 5: 0.70% | Year 0: 0.052%*  Year 1: 0.052%  Year 2: 0.052%  Year 3: 0.052%  Year 4: 0.052%  Year 5: 0.198% | Public Health Agency of Canada  Heart and Stroke Foundation of Canada |
| Stroke | Year 0: 0.025%*  Year 1: 0.025%  Year 2: 0.025%  Year 3: 0.025%  Year 4: 0.025%  Year 5: 0.065% | Year 0: 0.022%*  Year 1: 0.022%  Year 2: 0.022%  Year 3: 0.022%  Year 4: 0.022%  Year 5: 0.049% | Public Health Agency of Canada  Heart and Stroke Foundation of Canada |
| Fatal MI | 1.80% | 1.80% | RAMQ Database |
| Fatal stroke | 4.10% | 4.10% | RAMQ Database |

* The transition probabilities for the year 0 was the prevalence of the complications at 40 years old, which was the age at the enter of the model

RAMQ : *Régie de l’assurance maladie du Québec*
